# *catena*-Poly[[aqua­(1,10-phenanthroline)cadmium(II)]-μ-benzene-1,4-di­carboxyl­ato]

**DOI:** 10.1107/S1600536808041913

**Published:** 2008-12-17

**Authors:** Hongping Hu

**Affiliations:** a College of Science, North University of China, Taiyuan, Shanxi. 030051, People’s Republic of China

## Abstract

The title compound, [Cd(C_8_H_4_O_4_)(C_12_H_8_N_2_)(H_2_O)]_*n*_, is a new coordination polymer of benzene-1,4-dicarboxyl­ate with cadmium(II) and 1,10-phenanthroline. The Cd^II^ ion is coordinated by two N atoms from the 1,10-phenanthroline mol­ecule, three O atoms from two crystallographically independent benzene-1,4-dicarboxyl­ate ligands and the O atom of a coordinated water mol­ecule, forming a heavily distorted octa­hedron. The 1,10-phenanthroline ligand is approximately planar within 0.073 (4) Å. The two different benzene-1,4-dicarboxyl­ate ligands each coordinate to two Cd^II^ ions in bidentate and monodentate modes, forming an infinite zigzag chain. Adjacent chains are packed tightly by strong π–π inter­actions [centroid–centroid distances = 3.851 (2) and 3.859 (2) Å] between the aromatic rings of the benzene-1,4-dicarboxyl­ate ligand and the 1,10-phenanthroline of a neighboring chain, forming a sheet parallel to (011). Different sheets are linked together *via* O—H⋯O hydrogen bonds between the coordinated water mol­ecules and the O atoms of the carboxyl­ate groups, forming a three-dimensional network.

## Related literature

For related literature, see: Go *et al.* (2004 ▶); Sun *et al.* (2001 ▶). 
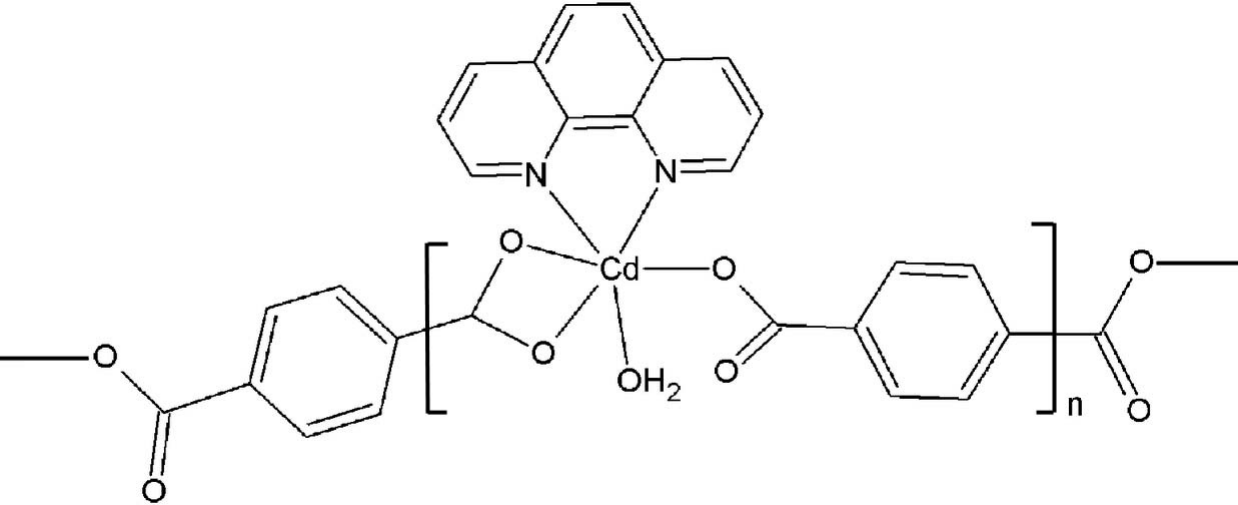


## Experimental

### 

#### Crystal data

[Cd(C_8_H_4_O_4_)(C_12_H_8_N_2_)(H_2_O)]
*M* *_r_* = 474.74
Triclinic, 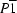

*a* = 9.1831 (5) Å
*b* = 9.6550 (6) Å
*c* = 11.3600 (7) Å
α = 104.6310 (8)°
β = 104.0390 (9)°
γ = 101.8920 (7)°
*V* = 906.28 (9) Å^3^
*Z* = 2
Mo *K*α radiation
μ = 1.24 mm^−1^
*T* = 298 (2) K
0.10 × 0.08 × 0.04 mm

#### Data collection

Bruker SMART CCD area-detector diffractometer
Absorption correction: multi-scan (*SADABS*; Sheldrick, 1996 ▶) *T*_min_ = 0.888, *T*_max_ = 0.952
5387 measured reflections
3939 independent reflections
3428 reflections with *I* > 2σ(*I*)
*R*_int_ = 0.018

#### Refinement

*R*[*F* ^2^ > 2σ(*F* ^2^)] = 0.033
*wR*(*F* ^2^) = 0.081
*S* = 1.07
3939 reflections
261 parameters
H atoms treated by a mixture of independent and constrained refinement
Δρ_max_ = 0.44 e Å^−3^
Δρ_min_ = −0.49 e Å^−3^

### 

Data collection: *SMART* (Bruker, 2007 ▶); cell refinement: *SAINT-Plus* (Bruker, 2007 ▶); data reduction: *SAINT-Plus*; program(s) used to solve structure: *SHELXS97* (Sheldrick, 2008 ▶); program(s) used to refine structure: *SHELXL97* (Sheldrick, 2008 ▶); molecular graphics: *SHELXTL* (Sheldrick, 2008 ▶); software used to prepare material for publication: *SHELXTL*.

## Supplementary Material

Crystal structure: contains datablocks global, I. DOI: 10.1107/S1600536808041913/ez2152sup1.cif

Structure factors: contains datablocks I. DOI: 10.1107/S1600536808041913/ez2152Isup2.hkl

Additional supplementary materials:  crystallographic information; 3D view; checkCIF report

## Figures and Tables

**Table 1:** Hydrogen-bond geometry (Å, °)

*D*—H⋯*A*	*D*—H	H⋯*A*	*D*⋯*A*	*D*—H⋯*A*
O5—H5*A*⋯O1^i^	0.81 (4)	1.91 (4)	2.697 (4)	163 (4)
O5—H5*B*⋯O4^ii^	0.75 (4)	2.07 (4)	2.782 (4)	159 (4)

Symmetry codes: (i) 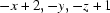; (ii) 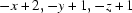.

**Fig. 1.**
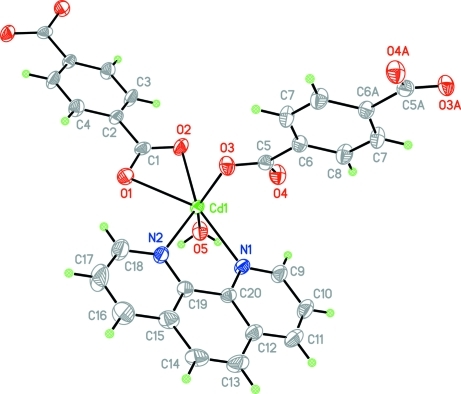
Molecular structure showing 50% probability displacement ellipsoids. Atoms marked with A and B are at the symmetry positions of (1 - x, -y, 1 - z) and (2 - x, 1 - y, -z), respectively.

**Fig. 2.**
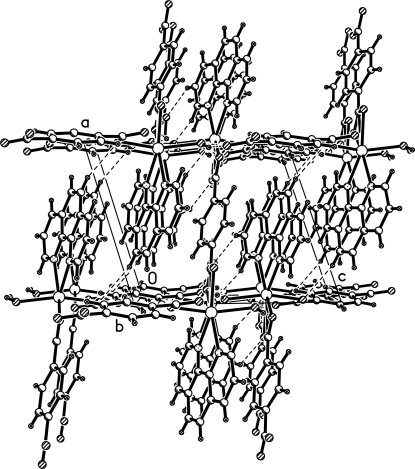
Packing diagram viewed down the b axis, The hydrogen bonds are indicated in dotted line.

## supplementary crystallographic information

### Comment

The title compound (I) was obtained by chance when the synthesis of its polymorph RAMJAQ (Sun *et al.*, 2001) was repeated for a fluorescence study. A single-crystal suitable for X-ray diffraction of I was crystallized from an H_2_O-EtOH (1:1) solvent mixture at room temperature.

The Cd^II^ ion is coordinated by two N atoms from the 1,10-phenanthroline, three O atoms from two crystallographically independent benzene-1,4-dicarboxylate ligands, and one O atom of a water molecule (Fig. 1). The 1,10-phenanthroline ligand is approximately planar, the maximum deviation of the C10 atom from the mean plane being 0.073 (4) Å. The geometries of the two crystallographically independent benzene-1,4-dicarboxylate ligands in (I) are similar to those observed by Go *et al.* (2004). The two different benzene-1,4-dicarboxylate ligands each coordinate to two Cd^II^ ions in chelate bidentate and monodentate modes, respectively, forming an infinite zigzag chain. All the bond distances and bond angles in the ligand are comparable to those values in its polymorph (Sun *et al.*, 2001). Adjacent chains are packed tightly by strong π-π interactions between the aromatic rings of the 1,10-phenanthroline and benzene-1,4-dicarboxylate ligands to form a sheet along the (011) direction. Strong π-π interactions between the aromatic rings are indicated by the short distance between C2 and C12 of 3.580 (6) Å. Different sheets are linked together though hydrogen bonds (Table 1) between coordinated the water molecules and O atoms of the carboxylate groups to form a three-dimensional network (Fig. 2).

### Experimental

Cd(NO_3_)_2_.4H_2_O (0.5 mmol, 154 mg), benzene-1,4-dicarboxylate acid (0.5 mmol, 84 mg), and 1,10-phenanthroline (0.5 mmol 90 mg) were added into 30 ml of the mixed solvent water and EtOH (1:1). The mixture was stirred at room temperature for 30 minutes and the pH value was adjusted to 7 by 1*M* NaOH to get a clear solution. The solution was allowed to evaporate in the air. Plate crystals of the title compound were obtained after 2 days. The crystals were filtered, washed by cold MeOH and dried in air. Crystals of (I) suitable for single-crystal X-ray diffraction were selected directly from the sample as prepared.

### Refinement

H atoms bonded to atom O5 were located in a difference map and refined without any restraints. Other H atoms were positioned geometrically and refined using a riding model with C—H = 0.93 (2) Å, and *U*_iso_(H) = 1.2 times *U*_eq_(C).

### Crystal data

**Table:**

[Cd(C_8_H_4_O_4_)(C_12_H_8_N_2_)(H_2_O)]	*Z* = 2
*M**_r_* = 474.74	*F*(000) = 472
Triclinic, *P*1	*D*_x_ = 1.74 Mg m^−^^3^
Hall symbol: -P 1	Mo *K*α radiation, λ = 0.71073 Å
*a* = 9.1831 (5) Å	Cell parameters from 2096 reflections
*b* = 9.6550 (6) Å	θ = 2.6–26.7°
*c* = 11.3600 (7) Å	µ = 1.24 mm^−^^1^
α = 104.6310 (8)°	*T* = 298 K
β = 104.0390 (9)°	Plate, colourless
γ = 101.8920 (7)°	0.10 × 0.08 × 0.04 mm
*V* = 906.28 (9) Å^3^	

### Data collection

**Table:**

Bruker SMART CCD area-detector diffractometer	3939 independent reflections
Radiation source: fine-focus sealed tube	3428 reflections with *I* > 2σ(*I*)
graphite	*R*_int_ = 0.018
φ and ω scans	θ_max_ = 27.5°, θ_min_ = 2.0°
Absorption correction: multi-scan (*SADABS*; Sheldrick, 1996)	*h* = −8→11
*T*_min_ = 0.888, *T*_max_ = 0.952	*k* = −11→12
5387 measured reflections	*l* = −14→13

### Refinement

**Table:**

Refinement on *F*^2^	Primary atom site location: structure-invariant direct methods
Least-squares matrix: full	Secondary atom site location: difference Fourier map
*R*[*F*^2^ > 2σ(*F*^2^)] = 0.033	Hydrogen site location: inferred from neighbouring sites
*wR*(*F*^2^) = 0.081	H atoms treated by a mixture of independent and constrained refinement
*S* = 1.07	*w* = 1/[σ^2^(*F*_o_^2^) + (0.0384*P*)^2^ + 0.2384*P*] where *P* = (*F*_o_^2^ + 2*F*_c_^2^)/3
3939 reflections	(Δ/σ)_max_ < 0.001
261 parameters	Δρ_max_ = 0.44 e Å^−^^3^
0 restraints	Δρ_min_ = −0.49 e Å^−^^3^

### Special details

**Table:**

Experimental. all of H atoms on water molecules were located on intermediate difference Fourier map
Geometry. All e.s.d.'s (except the e.s.d. in the dihedral angle between two l.s. planes) are estimated using the full covariance matrix. The cell e.s.d.'s are taken into account individually in the estimation of e.s.d.'s in distances, angles and torsion angles; correlations between e.s.d.'s in cell parameters are only used when they are defined by crystal symmetry. An approximate (isotropic) treatment of cell e.s.d.'s is used for estimating e.s.d.'s involving l.s. planes.
Refinement. Refinement of *F*^2^ against ALL reflections. The weighted *R*-factor *wR* and goodness of fit *S* are based on *F*^2^, conventional *R*-factors *R* are based on *F*, with *F* set to zero for negative *F*^2^. The threshold expression of *F*^2^ > σ(*F*^2^) is used only for calculating *R*-factors(gt) *etc*. and is not relevant to the choice of reflections for refinement. *R*-factors based on *F*^2^ are statistically about twice as large as those based on *F*, and *R*- factors based on ALL data will be even larger.

### Fractional atomic coordinates and isotropic or equivalent isotropic displacement parameters (Å^2^)

**Table:**

	*x*	*y*	*z*	*U*_iso_*/*U*_eq_	
Cd1	0.95793 (3)	0.18405 (3)	0.35302 (2)	0.02519 (9)	
O1	1.0863 (3)	−0.0176 (3)	0.3642 (3)	0.0414 (6)	
O2	1.2261 (3)	0.2183 (3)	0.4339 (3)	0.0405 (6)	
O3	0.9956 (3)	0.2355 (3)	0.1778 (2)	0.0359 (6)	
O4	1.0395 (3)	0.4563 (3)	0.3186 (2)	0.0391 (6)	
O5	0.9890 (4)	0.2639 (4)	0.5669 (3)	0.0405 (7)	
N1	0.7280 (3)	0.2663 (3)	0.3305 (3)	0.0303 (6)	
N2	0.7334 (3)	−0.0205 (3)	0.2344 (3)	0.0325 (7)	
C1	1.2161 (4)	0.0827 (4)	0.4162 (3)	0.0309 (8)	
C2	1.3631 (4)	0.0395 (4)	0.4593 (3)	0.0301 (7)	
C3	1.4937 (4)	0.1431 (4)	0.5520 (4)	0.0380 (9)	
H3	1.4903	0.2399	0.5871	0.046*	
C4	1.3706 (4)	−0.1043 (4)	0.4069 (4)	0.0374 (9)	
H4	1.2838	−0.1749	0.3439	0.045*	
C5	1.0181 (4)	0.3738 (4)	0.2075 (3)	0.0300 (7)	
C6	1.0122 (4)	0.4413 (4)	0.1011 (3)	0.0291 (7)	
C7	1.0364 (5)	0.3674 (4)	−0.0097 (3)	0.0373 (9)	
H7	1.0609	0.2775	−0.0170	0.045*	
C8	0.9751 (5)	0.5750 (4)	0.1105 (4)	0.0398 (9)	
H8	0.9580	0.6262	0.1847	0.048*	
C9	0.7232 (5)	0.4041 (4)	0.3839 (4)	0.0401 (9)	
H9	0.8158	0.4753	0.4379	0.048*	
C10	0.5850 (5)	0.4462 (5)	0.3622 (4)	0.0499 (11)	
H10	0.5859	0.5431	0.4027	0.060*	
C11	0.4501 (5)	0.3451 (5)	0.2821 (4)	0.0499 (11)	
H11	0.3579	0.3728	0.2663	0.060*	
C12	0.4487 (4)	0.1981 (4)	0.2224 (4)	0.0362 (8)	
C13	0.3116 (4)	0.0852 (5)	0.1363 (4)	0.0475 (10)	
H13	0.2174	0.1088	0.1161	0.057*	
C14	0.3154 (4)	−0.0539 (5)	0.0839 (4)	0.0476 (10)	
H14	0.2250	−0.1247	0.0258	0.057*	
C15	0.4574 (4)	−0.0949 (4)	0.1162 (4)	0.0418 (9)	
C16	0.4649 (5)	−0.2426 (5)	0.0711 (5)	0.0592 (13)	
H16	0.3758	−0.3178	0.0162	0.071*	
C17	0.6024 (6)	−0.2744 (5)	0.1081 (6)	0.0740 (17)	
H17	0.6083	−0.3716	0.0795	0.089*	
C18	0.7343 (5)	−0.1603 (4)	0.1893 (4)	0.0507 (11)	
H18	0.8282	−0.1835	0.2131	0.061*	
C19	0.5959 (4)	0.0121 (4)	0.1992 (3)	0.0295 (7)	
C20	0.5924 (4)	0.1633 (4)	0.2516 (3)	0.0274 (7)	
H5A	0.958 (5)	0.200 (4)	0.596 (4)	0.032 (11)*	
H5B	0.960 (5)	0.327 (4)	0.596 (4)	0.035 (13)*	

### Atomic displacement parameters (Å^2^)

**Table:**

	*U*^11^	*U*^22^	*U*^33^	*U*^12^	*U*^13^	*U*^23^
Cd1	0.02248 (13)	0.02768 (14)	0.02752 (14)	0.01040 (9)	0.00638 (9)	0.01107 (10)
O1	0.0258 (13)	0.0428 (15)	0.0577 (17)	0.0128 (12)	0.0060 (12)	0.0240 (13)
O2	0.0316 (14)	0.0378 (15)	0.0560 (17)	0.0191 (12)	0.0097 (12)	0.0180 (13)
O3	0.0457 (15)	0.0339 (14)	0.0333 (13)	0.0135 (12)	0.0120 (12)	0.0178 (11)
O4	0.0536 (17)	0.0383 (14)	0.0279 (13)	0.0153 (13)	0.0120 (12)	0.0136 (11)
O5	0.0595 (19)	0.0346 (16)	0.0337 (15)	0.0177 (15)	0.0206 (14)	0.0124 (13)
N1	0.0245 (15)	0.0288 (15)	0.0336 (15)	0.0099 (12)	0.0050 (12)	0.0054 (13)
N2	0.0275 (15)	0.0264 (15)	0.0388 (16)	0.0091 (12)	0.0055 (13)	0.0058 (13)
C1	0.0243 (17)	0.045 (2)	0.0348 (18)	0.0181 (16)	0.0120 (15)	0.0218 (16)
C2	0.0251 (17)	0.0342 (19)	0.0364 (19)	0.0156 (15)	0.0090 (15)	0.0151 (15)
C3	0.0314 (19)	0.0314 (19)	0.049 (2)	0.0189 (16)	0.0052 (17)	0.0081 (17)
C4	0.0283 (19)	0.0334 (19)	0.043 (2)	0.0106 (16)	0.0010 (16)	0.0080 (16)
C5	0.0274 (17)	0.0364 (19)	0.0305 (18)	0.0120 (15)	0.0090 (14)	0.0154 (15)
C6	0.0308 (18)	0.0300 (18)	0.0267 (17)	0.0109 (15)	0.0066 (14)	0.0102 (14)
C7	0.056 (2)	0.0310 (19)	0.0315 (19)	0.0223 (18)	0.0147 (17)	0.0124 (15)
C8	0.057 (3)	0.041 (2)	0.0315 (19)	0.0259 (19)	0.0199 (18)	0.0137 (16)
C9	0.035 (2)	0.0285 (19)	0.050 (2)	0.0104 (16)	0.0091 (18)	0.0039 (17)
C10	0.047 (2)	0.034 (2)	0.070 (3)	0.0220 (19)	0.019 (2)	0.010 (2)
C11	0.035 (2)	0.051 (3)	0.069 (3)	0.024 (2)	0.015 (2)	0.019 (2)
C12	0.0273 (18)	0.040 (2)	0.042 (2)	0.0138 (16)	0.0068 (16)	0.0136 (17)
C13	0.0243 (19)	0.059 (3)	0.052 (2)	0.0127 (19)	0.0004 (18)	0.017 (2)
C14	0.0206 (18)	0.053 (3)	0.049 (2)	−0.0023 (17)	−0.0048 (17)	0.009 (2)
C15	0.032 (2)	0.043 (2)	0.041 (2)	0.0043 (17)	0.0055 (17)	0.0069 (18)
C16	0.041 (2)	0.040 (2)	0.067 (3)	−0.002 (2)	−0.002 (2)	−0.005 (2)
C17	0.058 (3)	0.030 (2)	0.101 (4)	0.011 (2)	−0.003 (3)	−0.008 (2)
C18	0.039 (2)	0.034 (2)	0.067 (3)	0.0129 (18)	0.004 (2)	0.005 (2)
C19	0.0256 (17)	0.0290 (18)	0.0283 (17)	0.0054 (14)	0.0030 (14)	0.0071 (14)
C20	0.0234 (16)	0.0320 (18)	0.0273 (17)	0.0091 (14)	0.0065 (13)	0.0104 (14)

### Geometric parameters (Å, °)

**Table:**

Cd1—O3	2.256 (2)	C6—C8	1.387 (5)
Cd1—O5	2.281 (3)	C7—C8^ii^	1.385 (5)
Cd1—O2	2.330 (2)	C7—H7	0.9300
Cd1—N2	2.366 (3)	C8—C7^ii^	1.386 (5)
Cd1—N1	2.384 (3)	C8—H8	0.9300
Cd1—O1	2.489 (2)	C9—C10	1.396 (5)
O1—C1	1.265 (4)	C9—H9	0.9300
O2—C1	1.253 (4)	C10—C11	1.349 (6)
O3—C5	1.251 (4)	C10—H10	0.9300
O4—C5	1.255 (4)	C11—C12	1.406 (5)
O5—H5A	0.81 (4)	C11—H11	0.9300
O5—H5B	0.75 (4)	C12—C20	1.411 (5)
N1—C9	1.330 (4)	C12—C13	1.424 (5)
N1—C20	1.357 (4)	C13—C14	1.336 (6)
N2—C18	1.320 (5)	C13—H13	0.9300
N2—C19	1.354 (4)	C14—C15	1.430 (5)
C1—C2	1.501 (4)	C14—H14	0.9300
C2—C3	1.382 (5)	C15—C19	1.400 (5)
C2—C4	1.389 (5)	C15—C16	1.410 (6)
C3—C4^i^	1.382 (5)	C16—C17	1.354 (6)
C3—H3	0.9300	C16—H16	0.9300
C4—C3^i^	1.382 (5)	C17—C18	1.388 (6)
C4—H4	0.9300	C17—H17	0.9300
C5—C6	1.507 (5)	C18—H18	0.9300
C6—C7	1.374 (5)	C19—C20	1.441 (5)
			
O3—Cd1—O5	149.20 (11)	C8—C6—C5	120.8 (3)
O3—Cd1—O2	89.18 (9)	C6—C7—C8^ii^	121.0 (3)
O5—Cd1—O2	80.46 (10)	C6—C7—H7	119.5
O3—Cd1—N2	93.93 (10)	C8^ii^—C7—H7	119.5
O5—Cd1—N2	113.58 (11)	C7^ii^—C8—C6	120.1 (3)
O2—Cd1—N2	136.37 (9)	C7^ii^—C8—H8	119.9
O3—Cd1—N1	92.49 (10)	C6—C8—H8	119.9
O5—Cd1—N1	84.71 (10)	N1—C9—C10	122.7 (4)
O2—Cd1—N1	153.45 (10)	N1—C9—H9	118.6
N2—Cd1—N1	69.96 (9)	C10—C9—H9	118.6
O3—Cd1—O1	102.64 (9)	C11—C10—C9	119.5 (4)
O5—Cd1—O1	94.69 (10)	C11—C10—H10	120.3
O2—Cd1—O1	54.27 (9)	C9—C10—H10	120.3
N2—Cd1—O1	82.74 (9)	C10—C11—C12	120.2 (4)
N1—Cd1—O1	149.65 (9)	C10—C11—H11	119.9
C1—O1—Cd1	88.0 (2)	C12—C11—H11	119.9
C1—O2—Cd1	95.7 (2)	C11—C12—C20	117.0 (3)
C5—O3—Cd1	103.3 (2)	C11—C12—C13	123.6 (3)
Cd1—O5—H5A	116 (3)	C20—C12—C13	119.4 (3)
Cd1—O5—H5B	122 (3)	C14—C13—C12	121.4 (4)
H5A—O5—H5B	104 (4)	C14—C13—H13	119.3
C9—N1—C20	118.2 (3)	C12—C13—H13	119.3
C9—N1—Cd1	125.8 (2)	C13—C14—C15	120.6 (4)
C20—N1—Cd1	116.0 (2)	C13—C14—H14	119.7
C18—N2—C19	117.8 (3)	C15—C14—H14	119.7
C18—N2—Cd1	125.2 (3)	C19—C15—C16	116.9 (4)
C19—N2—Cd1	116.6 (2)	C19—C15—C14	120.3 (4)
O2—C1—O1	122.0 (3)	C16—C15—C14	122.8 (4)
O2—C1—C2	118.5 (3)	C17—C16—C15	119.8 (4)
O1—C1—C2	119.5 (3)	C17—C16—H16	120.1
C3—C2—C4	118.9 (3)	C15—C16—H16	120.1
C3—C2—C1	120.2 (3)	C16—C17—C18	119.2 (4)
C4—C2—C1	120.9 (3)	C16—C17—H17	120.4
C2—C3—C4^i^	120.7 (3)	C18—C17—H17	120.4
C2—C3—H3	119.6	N2—C18—C17	123.4 (4)
C4^i^—C3—H3	119.6	N2—C18—H18	118.3
C3^i^—C4—C2	120.3 (3)	C17—C18—H18	118.3
C3^i^—C4—H4	119.8	N2—C19—C15	122.9 (3)
C2—C4—H4	119.8	N2—C19—C20	118.2 (3)
O3—C5—O4	123.6 (3)	C15—C19—C20	118.9 (3)
O3—C5—C6	117.0 (3)	N1—C20—C12	122.4 (3)
O4—C5—C6	119.3 (3)	N1—C20—C19	118.2 (3)
C7—C6—C8	118.9 (3)	C12—C20—C19	119.3 (3)
C7—C6—C5	120.3 (3)		
			
O3—Cd1—O1—C1	80.9 (2)	Cd1—C1—C2—C3	−7(3)
O5—Cd1—O1—C1	−73.5 (2)	O2—C1—C2—C4	−158.3 (4)
O2—Cd1—O1—C1	1.20 (19)	O1—C1—C2—C4	22.1 (5)
N2—Cd1—O1—C1	173.3 (2)	Cd1—C1—C2—C4	173 (3)
N1—Cd1—O1—C1	−161.0 (2)	C4—C2—C3—C4^i^	−0.6 (6)
O3—Cd1—O2—C1	−107.5 (2)	C1—C2—C3—C4^i^	179.6 (3)
O5—Cd1—O2—C1	101.7 (2)	C3—C2—C4—C3^i^	0.6 (6)
N2—Cd1—O2—C1	−12.6 (3)	C1—C2—C4—C3^i^	−179.6 (3)
N1—Cd1—O2—C1	158.6 (2)	Cd1—O3—C5—O4	10.6 (4)
O1—Cd1—O2—C1	−1.2 (2)	Cd1—O3—C5—C6	−166.9 (2)
O5—Cd1—O3—C5	−20.9 (3)	O3—C5—C6—C7	−22.1 (5)
O2—Cd1—O3—C5	−90.5 (2)	O4—C5—C6—C7	160.3 (3)
N2—Cd1—O3—C5	133.0 (2)	O3—C5—C6—C8	154.9 (4)
N1—Cd1—O3—C5	62.9 (2)	O4—C5—C6—C8	−22.7 (5)
O1—Cd1—O3—C5	−143.6 (2)	C8—C6—C7—C8^ii^	0.2 (7)
C1—Cd1—O3—C5	−116.4 (2)	C5—C6—C7—C8^ii^	177.3 (4)
O3—Cd1—N1—C9	−91.3 (3)	C7—C6—C8—C7^ii^	−0.2 (7)
O5—Cd1—N1—C9	57.9 (3)	C5—C6—C8—C7^ii^	−177.2 (3)
O2—Cd1—N1—C9	1.8 (4)	C20—N1—C9—C10	0.2 (6)
N2—Cd1—N1—C9	175.4 (3)	Cd1—N1—C9—C10	177.2 (3)
O1—Cd1—N1—C9	148.2 (3)	N1—C9—C10—C11	−1.3 (7)
C1—Cd1—N1—C9	84.5 (7)	C9—C10—C11—C12	0.9 (7)
O3—Cd1—N1—C20	85.8 (2)	C10—C11—C12—C20	0.6 (6)
O5—Cd1—N1—C20	−125.0 (2)	C10—C11—C12—C13	−179.7 (4)
O2—Cd1—N1—C20	178.9 (2)	C11—C12—C13—C14	−178.6 (4)
N2—Cd1—N1—C20	−7.5 (2)	C20—C12—C13—C14	1.0 (6)
O1—Cd1—N1—C20	−34.7 (3)	C12—C13—C14—C15	2.0 (7)
C1—Cd1—N1—C20	−98.4 (6)	C13—C14—C15—C19	−2.7 (7)
O3—Cd1—N2—C18	90.0 (3)	C13—C14—C15—C16	175.1 (5)
O5—Cd1—N2—C18	−104.2 (3)	C19—C15—C16—C17	−0.4 (7)
O2—Cd1—N2—C18	−2.9 (4)	C14—C15—C16—C17	−178.3 (5)
N1—Cd1—N2—C18	−178.7 (4)	C15—C16—C17—C18	−0.6 (9)
O1—Cd1—N2—C18	−12.2 (3)	C19—N2—C18—C17	0.1 (7)
C1—Cd1—N2—C18	−8.9 (4)	Cd1—N2—C18—C17	−172.5 (4)
O3—Cd1—N2—C19	−82.7 (3)	C16—C17—C18—N2	0.8 (9)
O5—Cd1—N2—C19	83.1 (3)	C18—N2—C19—C15	−1.2 (6)
O2—Cd1—N2—C19	−175.6 (2)	Cd1—N2—C19—C15	172.1 (3)
N1—Cd1—N2—C19	8.6 (2)	C18—N2—C19—C20	177.8 (4)
O1—Cd1—N2—C19	175.1 (3)	Cd1—N2—C19—C20	−9.0 (4)
C1—Cd1—N2—C19	178.4 (2)	C16—C15—C19—N2	1.3 (6)
Cd1—O2—C1—O1	2.3 (4)	C14—C15—C19—N2	179.2 (4)
Cd1—O2—C1—C2	−177.3 (3)	C16—C15—C19—C20	−177.6 (4)
Cd1—O1—C1—O2	−2.1 (3)	C14—C15—C19—C20	0.3 (6)
Cd1—O1—C1—C2	177.5 (3)	C9—N1—C20—C12	1.5 (5)
O3—Cd1—C1—O2	74.0 (2)	Cd1—N1—C20—C12	−175.9 (3)
O5—Cd1—C1—O2	−75.3 (2)	C9—N1—C20—C19	−176.7 (3)
N2—Cd1—C1—O2	170.8 (2)	Cd1—N1—C20—C19	6.0 (4)
N1—Cd1—C1—O2	−101.9 (6)	C11—C12—C20—N1	−1.9 (5)
O1—Cd1—C1—O2	177.8 (3)	C13—C12—C20—N1	178.5 (3)
O3—Cd1—C1—O1	−103.9 (2)	C11—C12—C20—C19	176.3 (3)
O5—Cd1—C1—O1	106.8 (2)	C13—C12—C20—C19	−3.4 (5)
O2—Cd1—C1—O1	−177.8 (3)	N2—C19—C20—N1	1.9 (5)
N2—Cd1—C1—O1	−7.1 (2)	C15—C19—C20—N1	−179.1 (3)
N1—Cd1—C1—O1	80.3 (6)	N2—C19—C20—C12	−176.3 (3)
O2—C1—C2—C3	21.6 (5)	C15—C19—C20—C12	2.7 (5)
O1—C1—C2—C3	−158.0 (4)		

Symmetry codes: (i) −*x*+3, −*y*, −*z*+1; (ii) −*x*+2, −*y*+1, −*z*.

### Hydrogen-bond geometry (Å, °)

**Table:**

*D*—H···*A*	*D*—H	H···*A*	*D*···*A*	*D*—H···*A*
O5—H5A···O1^iii^	0.81 (4)	1.91 (4)	2.697 (4)	163 (4)
O5—H5B···O4^iv^	0.75 (4)	2.07 (4)	2.782 (4)	159 (4)

Symmetry codes: (iii) −*x*+2, −*y*, −*z*+1; (iv) −*x*+2, −*y*+1, −*z*+1.
